# The Effect of the First Coronavirus Lockdown on Psychiatric Outpatient Attendance, a North Fife Survey

**DOI:** 10.1192/bjo.2022.422

**Published:** 2022-06-20

**Authors:** Adebola Adegbite, George Howson

**Affiliations:** 1 NHS Scotland, Fife, United Kingdom; 2 NHS Scotland, Edinburgh, United Kingdom

## Abstract

**Aims:**

There has been a significant change in the way we see patients during psychiatric consultations, this has led to challenges we face in delivering safe and effective care to patients under our care. “Telepsychiatry” has been used in literature from countries like Australia and India, there is very little around coming from the UK but there appears to be many ongoing research making the rounds. It is interesting to know that the existing literature on remote/virtual consultations during the COVID-19 pandemic are on the rise. The idea of this study was conceived during outpatient clinics after making an observation that many patients were likely to miss their appointments when they had telephone appointments compared to video consultations. This prompted a study to know if this is more likely to be observed in other outpatient clinics. The purpose of this study was to establish if virtual/remote consulting has affected patient attendance rate and whether this is also affected by the type of virtual consultation.

**Methods:**

The data were collected using the “2020 stats sheets” for inpatient appointments between North Fife consultants from January to October 2020. This was registered with the NHS Fife clinical effectiveness team in January 2021.

**Results:**

The results were categorized for the purpose of this survey as January – March (Pre-lockdown) and April – October (lockdown). It is important to note that some face-to-face appointments occurred during lockdown because there were emergency assessments and drug monitoring appointments scheduled.

The results of this survey showed that there was a clear reduction in clinic appointments made during lockdown compared to pre-lockdown and slight observable improvement in attendance rates during the lockdown. There was no statistical significance seen using t-test comparing attendance rates between video and telephone consultations including new patient virtual consultations.

**Conclusion:**

The large sample size over this period suggests that the results are reliable and valid, we can therefore say virtual/telephone consultation does not affect attendance. It should be noted that the attendance rate may be a good indicator but we should also consider patient/clinician satisfaction, communication quality/effectiveness and other factors which could influence patient's compliance to outpatient follow-up. It is important to acknowledge the lack of a control group and the COVID-19 pandemic were major cofounding factors. Mental health services should continue the use of virtual consultation post-pandemic and possibly integrate it with in person consultations (hybrid), this may help with attendance rate of patients with difficulty attending face-to-face appointments.

